# Appearance of the levator ani muscle subdivisions on 3D transperineal ultrasound

**DOI:** 10.1186/s13244-021-01037-y

**Published:** 2021-07-02

**Authors:** Claudia Manzini, Frieda van den Noort, Anique T. M. Grob, Mariëlla I. J. Withagen, Cornelis H. Slump, C. Huub van der Vaart

**Affiliations:** 1 Department of Obstetrics and Gynecology, University Medical Centre Utrecht, Utrecht, The Netherlands; 2 Robotics and Mechatronics, University of Twente, Enschede, Carre 3.526, Drienerlolaan 5, 7522NB Enschede, The Netherlands; 3 Multi-Modality Medical Imaging, Faculty of Science and Technology, Technical Medical Centre, University of Twente, Enschede, The Netherlands

**Keywords:** Levator ani muscle, Segmentation, Transperineal ultrasound, Pelvic floor

## Abstract

**Background:**

The levator ani muscle (LAM) consists of different subdivisions, which play a specific role in the pelvic floor mechanics. The aim of this study is to identify and describe the appearance of these subdivisions on 3-Dimensional (3D) transperineal ultrasound (TPUS). To do so, a study designed in three phases was performed in which twenty 3D TPUS scans of vaginally nulliparous women were assessed. The first phase was aimed at getting acquainted with the anatomy of the LAM subdivisions and its appearance on TPUS: relevant literature was consulted, and the TPUS scan of one patient was analyzed to identify the puborectal, iliococcygeal, puboperineal, pubovaginal, and puboanal muscle. In the second phase, the five LAM subdivisions and the pubic bone and external sphincter, used as reference structures, were manually segmented in volume data obtained from five nulliparous women at rest. In the third phase, intra- and inter-observer reproducibility were assessed on twenty TPUS scans by measuring the Dice Similarity Index (DSI).

**Results:**

The mean inter-observer and median intra-observer DSI values (with interquartile range) were: puborectal 0.83 (0.13)/0.83 (0.10), puboanal 0.70 (0.16)/0.79 (0.09), iliococcygeal 0.73 (0.14)/0.79 (0.10), puboperineal 0.63 (0.25)/0.75 (0.22), pubovaginal muscle 0.62 (0.22)/0.71 (0.16), and the external sphincter 0.81 (0.12)/0.89 (0.03).

**Conclusion:**

Our results show that the LAM subdivisions of nulliparous women can be reproducibly identified on 3D TPUS data.

**Supplementary Information:**

The online version contains supplementary material available at 10.1186/s13244-021-01037-y.

## Keypoints

- The levator ani muscle (LAM) plays a key role in pelvic floor (dys)function.
- The LAM consists of subdivisions which have specific functions.
- Transperineal ultrasound (TPUS) enables (functional) assessment of the LAM.
- LAM subdivisions can be identified on TPUS of women with intact LAM.
- This is the first step for TPUS-based biomechanical analysis of the LAM.

## Background

The prevalence of pelvic floor disorders is high [1, 2], and the long-term effectiveness of treatments relatively limited [3, 4]. This prompted DeLancey to publish a paper in 2005 in which a goal was set to achieve 25% reduction in occurrence and 25% improvement in treatment success by 2025 [5]. In 2017 it was acknowledged that measurable improvements were not yet achieved. However, the scientific community was (and is) gaining the quantitative framework necessary to spur the progress [6]. This quantitative framework includes pelvic floor (PF) biomechanical analyses [7–10], which allow us to get insight into PF functionality and understand the functional impact of PF damage. To perform biomechanical analyses, computer simulations and measurements are produced from image data. This implies that interpreting image data accurately is fundamental, if we want to draw meaningful conclusions. Moreover, the functional consequence of LAM injury may depend on the region of muscle affected [11]. To test this hypothesis in imaging studies, the different LAM regions (or subdivisions) have to be correctly identified, which prompted the current study.

The 3D appearance of the levator ani muscle (LAM) subdivisions of nulliparous women have been described on magnetic resonance imaging (MRI) and endovaginal ultrasound, respectively [12, 13]. To the best of our knowledge, this has not yet been achieved with transperineal ultrasound (TPUS). Compared to MRI, TPUS is less expensive and data acquisition is faster. The advantage is that a large dataset can be easier collected, providing statistically robust results. Differently from endovaginal ultrasound, TPUS can capture PF motion, thus providing the functional information that is necessary to validate biomechanical analyses [14].

TPUS is currently used in scientific research on and clinical assessment of PF disorders: it allows for the assessment of the anterior, apical and posterior compartment, LAM avulsion, anal sphincter, and implants materials [15–19]. In addition, TPUS has been applied for investigating the consequences of pregnancy and delivery on PF biometry and integrity [20–26]. Analyses and measurements are mostly performed in 2D.

In 2018 we have published a protocol for reproducible 3D segmentation [27] for the part of the LAM surrounding the hiatal area, without discriminating between the different LAM subdivisions. During the last years, advancements in TPUS hardware and software led to significant improvements in image quality. Therefore, the aim of this study is to identify and describe the separate appearance of LAM subdivisions on 3D TPUS of vaginally nulliparous women.

## Methods

The ultrasound data used for the present study were collected as a subset within the GYNecological Imaging using 3D UltraSound (GYNIUS) project on the assessment of pelvic floor contractility with TPUS, which is conducted at our tertiary urogynecological clinic. The data were acquired with a Philips Epiq 7G ultrasound machine connected to a X6-1 matrix transducer. The volume angle was 90° in both azimuthal and elevational direction and probe consists of 9212 elements. Post-processing filters were set off; the scan depth was 9 cm. The resolution of the image was 0.6 mm between the 229 sagittal slices, 0.4 mm between the 352 coronal slices and 0.3 mm between the 277 axial slices. In order to make the LAM fully visible within the coronal opening angle, the transducer was covered with a 2 cm thick gel pad, which created more distance between the patient and the probe. All scans were performed in supine position with empty bladder. Since the aim of our study was to describe the appearance of the LAM subdivisions of an intact pelvic floor, only vaginally nulliparous women were included. The Medical Research Ethics Committee (MREC) UMC Utrecht exempted the project from approval (reference 18/215), because the Medical Research Involving Human Subject Act (WMO) does not apply, and all women signed a research consent form.

We conducted the study in three phases. The first phase was aimed at gaining familiarity with the anatomy of the LAM subdivisions. Initially, we consulted relevant literature about the topic [12, 13, 28] to evaluate if the definition of the different subdivisions was consistent between authors in terms of nomenclature, shape, and orientation. Having done this, we aimed at identifying on TPUS the following LAM subdivisions: the puborectal muscle (PRM), iliococcygeal muscle (ICM) and pubovisceral muscle, the latter consisting of the puboperineal muscle (PPM), pubovaginal muscle (PVM), and puboanal muscle (PAM). For this purpose, one TPUS was analyzed and the five LAM subdivisions were identified using the following criteria [12]: a distinct and consistently visible separation between a structure and adjacent structures and/or differing origin or insertion of the muscle.

**Fig. 1:**
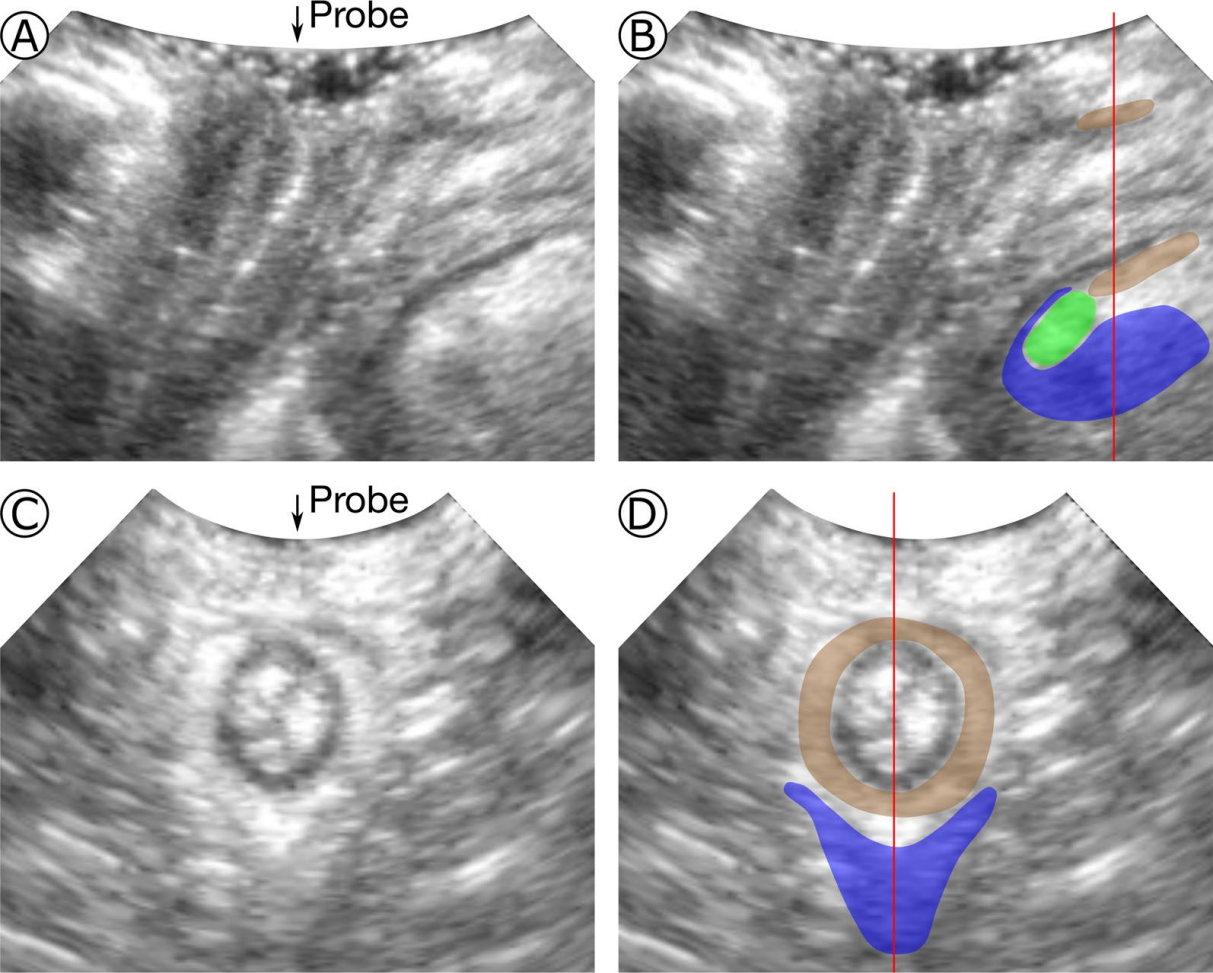
Midsagittal (**A** and **B**) and coronal (**C** and **D**) plane, without and with segmentation. In **B**, the C shape of the iliococcygeal muscle (ICM) (blue) surrounding the puborectal muscle (PRM) (green); in **D**, the round shape of the external sphincter (brown). The red line in **B** shows the position of the coronal plane and in **D**, the position of the midsagittal plane (**A** and **B**). The ultrasound probe position with respect to the images is indicated by the arrows

**Fig. 2:**
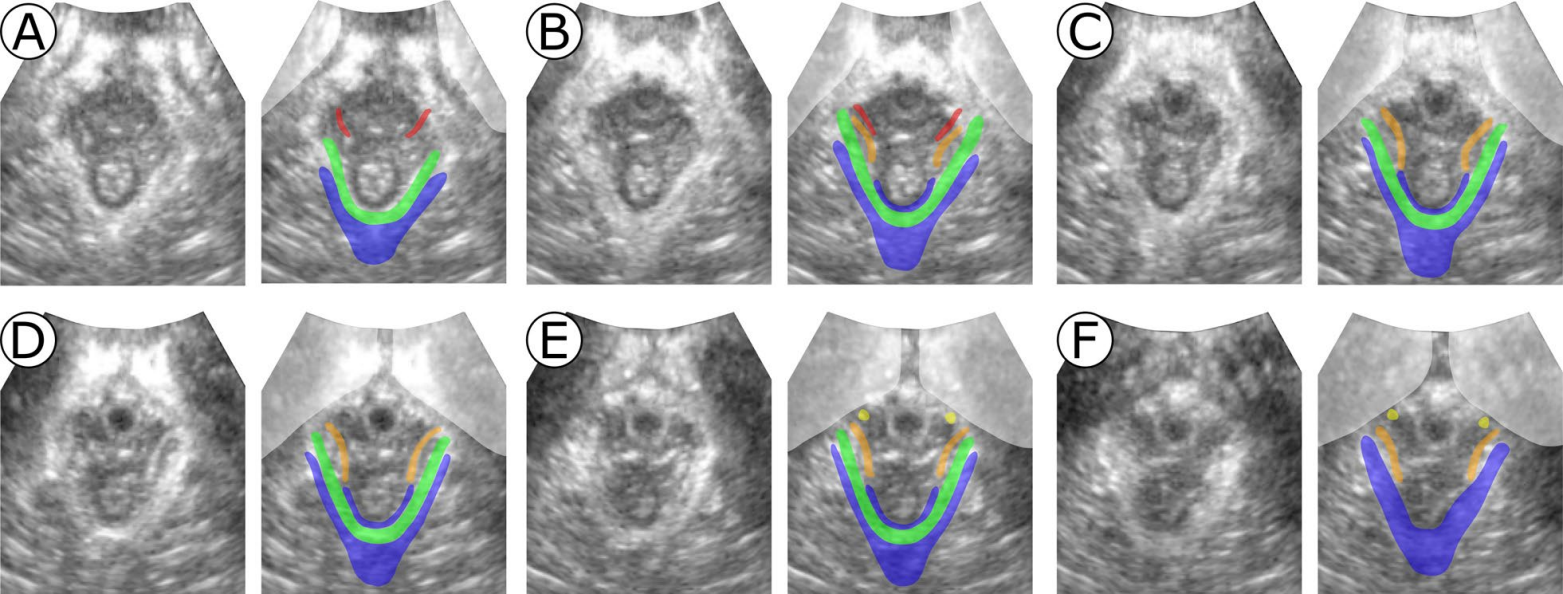
Slices parallel to the plane of minimal hiatal dimensions (2.4 mm between subsequent slices). The image without (left) and with segmentation (right) is displayed for each slice. From **A** to **F** the slices are ordered in the caudal-cranial direction. Slice **D** shows the plane of minimal hiatal dimensions. Segmented structures: pubic bone (PB, grey), puboperineal muscle (PPM, red), puboanal muscle (PAM, orange), pubovaginal muscle (PVM, yellow), puborectal muscle (PRM, green) and iliococcygeal muscle (ICM, blue)

In the second phase of the study, the pubic bone (PB) and external sphincter (ES), used as reference structures, and the LAM subdivisions were manually segmented on five TPUS scans. To perform the segmentations an in-house software was developed in MeVisLab [29] (Figs. 1 and 2). The following manual segmentation protocol was used:

- Reference structures, i.e., pubic bone (PB) and external sphincter (ES)

In order to have a ventral and dorsal reference the PB and ES were segmented first. Analyzing the TPUS volumes on the axial plane in the caudal-cranial direction, the PB is the first structure visualized, appearing hyperechoic at its boundaries and hypoechoic internally, which makes it easy to recognize and segment it. For a correct segmentation of the ES it is useful to identify its boundaries on the midsagittal plane, where its separation with the LAM appears as a hypoechoic line between two hyperechoic structures. Having done this, the coronal plane has to be rotated perpendicular to the anal canal. On this plane the ES appears as a hyperechoic circle which surrounds a hypoechoic circle, the internal anal sphincter [18] (Fig. 1).

- LAM subdivisions, i.e., the puboperineal muscle (PPM), puboanal muscle (PAM), puborectal muscle (PRM), iliococcygeal muscle (ICM), and pubovaginal muscle (PVM)

In the axial direction the most superficial LAM subdivision is the PPM, which is a symmetrical hyperechoic structure attaching ventrolaterally to the PB and dorsomedially to the area between anal canal and vagina, where the perineal body is located. To visualize the PAM, PRM and ICM, the axial plane must be rotated to the plane of minimal hiatal dimensions [30] (Fig. 2D). In this plane, from medial to lateral, the PAM, PRM, and ICM can be recognized as three symmetrical structures, separated by a hypoechoic line. The PAM is located lateral to the vagina, and attaches ventrally to the PB, and dorsally to the fibers of the ES. The PRM, located laterally to the PAM, attaches ventrally to the PB and passes dorsally behind the rectum. Cranially from the plane of minimal hiatal dimensions, the most lateral part of the LAM appears highly hyperechoic. Here the ICM attaches to the arcus tendineus levator ani (ATLA), a condensation of connective tissue coursing along the surface of the obturator internus muscle [31]. The ATLA cannot be separated from the ICM on TPUS. Therefore, they were segmented as a single structure. From the attachments to the ATLA the ICM courses in the direction of the coccyx, curving around the PRM. The PVM is a small hypoechoic symmetrical structure between the PB and the anterior lateral edges of the vagina, medial to the PAM (Fig. 2). The appearance of the PRM and ICM on the mid-sagittal plane, and of the PRM, ICM, and PAM on the coronal plane was used as a reference for the segmentation on the axial plane (Fig. 1).

The slice-by-slice 2D segmentations were used to produce 3D models in MeVisLab in order to visualize the structures in their entirety.

The third phase of the study aimed at assessing the reproducibility of the segmentation procedure. For this purpose, we use the five TPUS of the second phase plus 15 new TPUS. The following four slices were selected in the ultrasound volumes:

1. Minimal hiatal dimensions slice where the PAM, PRM and ICM are visible (Fig. 2D);
2. An axial slice showing the PPM (similar to Fig. 2A);
3. An axial slice with the PVM (similar to Fig. 2F);
4. A slice perpendicular to the anal canal where the circular structure of the ES is visualized (Fig. 1D).

**Fig. 3:**
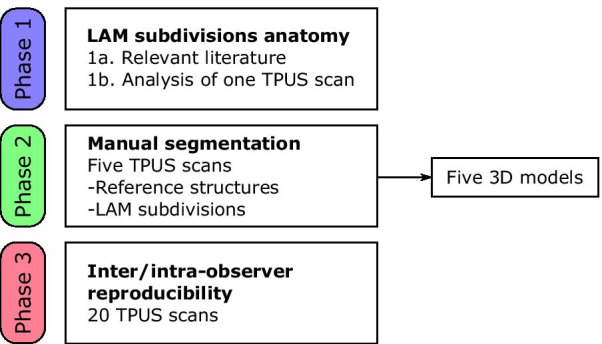
Flowchart showing the different study phases

C.M. and F.N. performed, independently, the segmentation of the four slices for all the 20 images, in order to assess inter-observer reproducibility. After more than one week, F.N. repeated all measurements on the 20 TPUS, segmented in a random order, to assess intra-observer reproducibility. To measure the intra- and interobserver overlap between segmentations, we use the Dice Similarity Index (DSI): DSI = 2(*X* ∩ *Y*)/(*X* + *Y*). This formula states that two times the overlapping area is divided by the sum of the area of segmentation *X* and segmentation *Y*; DSI = 0 corresponds to no overlap and DSI = 1 to maximum possible overlap. In Fig. 3 a flowchart summarizes the three study phases.

## Results

The 20 patients included in the study presented with symptoms of overactive pelvic floor confirmed by physical examination. The mean age was 39 years (range 19–68), and mean body mass index 22.7 (range 17.0–29.0). None of them had vaginally delivered before nor had any prior pelvic floor surgeries.

**Fig. 4:**
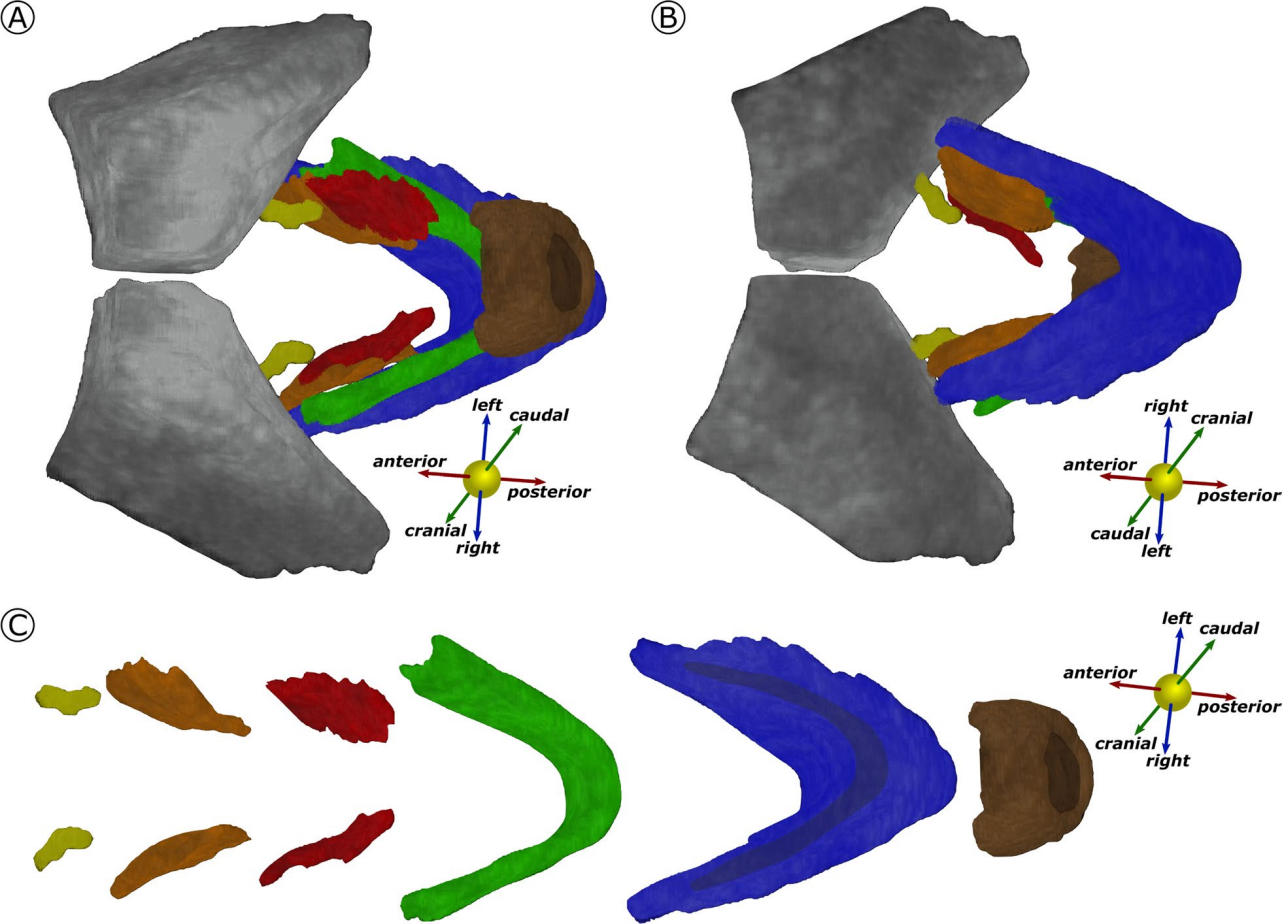
The 3D model showing the segmented structures: The Pubic bone (PB, grey), external sphincter (ES, brown), puboperineal muscle (PPM, red), puboanal muscle (PAM, orange), pubovaginal muscle (PVM, yellow), puborectal muscle (PRM, green) and iliococcygeal muscle (ICM, blue). **A** shows the view from caudal to cranial; **B** shows the view from cranial to caudal. In **C**, the model is disassembled to appreciate the single structures separately

From literature research and visual examination of TPUS data we were able to develop a manual segmentation protocol for the five LAM subdivisions. Applying our protocol, we were able to segment all LAM subdivisions in the five TPUS scans used for this purpose. The five 3D models (generated in MeVisLab) let appreciate the segmented structures in their entirety, their spatial direction, and the spatial relation between different structures (Fig. 4 and Appendix 1).

**Fig. 5:**
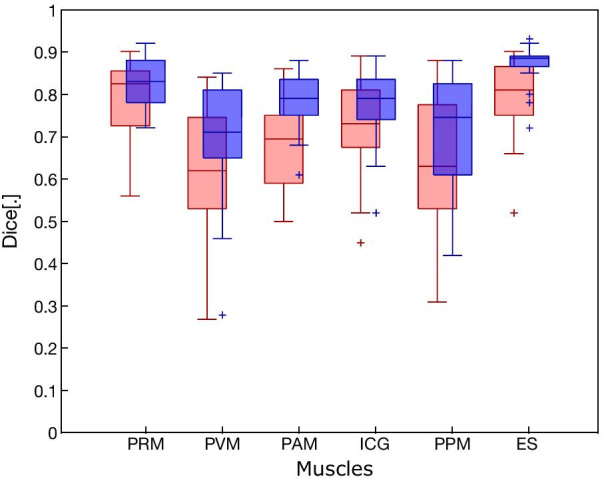
Box-and-whisker plots of Dice Similarity Index for inter-(red) and intraobserver (blue) overlap between segmentations of the puborectal (PRM), pubovaginal (PVM), puboanal (PAM), iliococcygeal (ICM) and puboperineal muscle (PPM) and external anal sphincter (ES). Boxes with internal lines represent median and interquartile range (IQR), whiskers are range excluding outliers more than 1.5 IQR from upper and lower quartile, and + are outliers

Figure 5 shows a Box and Whisker plot of the inter- and intraobserver DSI values of the 2D segmentations of 20 patients. All median DSI values of the ES, PRM, PAM and ICM were ≥ 0.7. In the case of intra-observer overlap, this was also true for the PPM and PVM. In the case of inter-observer overlap, the median DSI values of the PPM and PVM were below 0.7.

## Discussion

Our results demonstrate that the separate LAM muscle subdivisions can be identified in 3D TPUS images of vaginally nulliparous women, which has not been reported in literature.

In the literature, a DSI > 0.7 is described as excellent agreement [32, 33]. However, the DSI is influenced by the shape and size of the segmented structure: small and/or elongated structures are more likely to have a lower DSI, because a mismatch of a few pixels has relatively more influence. The DSI values of the PRM, PAM, ICM and ES show good segmentation reproducibility (comparable to previous results on the PRM [34]). The smallest structures (i.e., the PVM and PPM) are most of the time less than 5 mm thick. With voxel sizes of around 0.5 mm, a 1–2 voxels mismatch produces already a relatively large overlap mismatch, which can explain the lower DSI values. The DSI values of the PVM and PPM thus indicate that their identification is successful. However, in order to obtain reliable segmentation of these small structures a higher resolution would be needed.

Our study has several strengths. First, the 20 TPUS scans used were all acquired by the same clinician; thus, reducing a potential source of variability. Second, the segmentation protocol we have developed proved effective for all five TPUS scans used for this purpose. Third, to assess the reproducibility of our results, we measure the actual spatial overlap between different segmentations, i.e., a quantitative method, while in previous studies interrater reliability was assessed by evaluating whether a muscle was visible or not [12, 13]. Using this method, different observers can theoretically agree on the visibility of a muscle, while recognizing two different structures. This would result in 100% agreement when there is no actual agreement. This potential bias is avoided by calculating the actual spatial overlap between different segmentations.

Due to technical limitations, mainly related to resolution, we were unable to segment the most cranial structures of the PF, thus missing the upper border of the ICM and PAM. Additionally, we could not segment the most dorsal part of the PAM and PPM because of the presence of the perineal body in this area. Therefore, our 3D models may suggest that these muscles stop more ventrally than expected. However, the spatial relations between different LAM subdivisions can be fully appreciated.

Lastly, the same observers performed the first and third phase of the study (i.e., LAM subdivisions identification and assessment of segmentations reproducibility). One might thus object that the assessment of the reproducibility could have been biased towards higher scores. However, without prior identification of the structures of interest no reproducibility assessment is possible. In addition, the LAM subdivisions were reproducibly identified also on the TPUS never analyzed before the third phase of the study.

Currently TPUS data are analyzed in 2D, with the most important analysis method being the one developed by Dietz et al. [30]. Since TPUS can capture muscle movement in 3D, 2D analysis is a very low dimensional representation of the data. Our study opens the possibility to analyze static TPUS images in 3D. Additionally, having identified and segmented the different LAM subdivisions, TPUS-based biomechanical analyses can be applied on intact LAM. Das et al. [35] used the PRM segmentations from this study and successfully estimated 3D displacement and strain of the PRM, which has not been reported in literature before. These strain and displacement measurements provide a unique measurement of in vivo movement and function of the LAM and its subdivisions. This is the biggest advantage of TPUS over endovaginal ultrasound, because it is not possible to capture movement with endovaginal ultrasound. With respect to MRI, dynamic MRI does exist but it is much less available than TPUS. The work of Das et al. [35] demonstrates that our study is an important step in the direction of in vivo 3D biomechanical analysis of the pelvic floor function. This analysis could allow for a reliable quantitative assessment of the pelvic floor function to be used for diagnostic purposes and for the assessment of functional changes over time (e.g., during treatment).

Considering that the LAM subdivisions of women with normal pelvic organ support have different fiber directions, it was proposed that the functional consequence of LAM injury depends on the region of muscle affected [11]. Therefore, the appearance of LAM subdivisions on TPUS collected from vaginally nulliparous women can be used as a reference for studies in vaginally parous patients to identify selective damage to single pelvic floor structures. Shortly after the successful identification of the intact LAM subdivisions on MRI [12], Margulies et al. (36) analyzed 14 MRI scans of women with unilateral LAM defect and were able to identify the damaged portion as pubovisceral muscle. This shows that the ability to discriminate the intact LAM subdivisions allows for the recognition of the damaged LAM subdivisions. The same study, focusing on LAM damage, is to be replicated on TPUS and extended with in vivo muscle strain and displacement measurements. If successful, TPUS-based biomechanical analyses could be then performed to understand the functional consequences of this and other types of damage and, eventually, implement appropriate treatment strategies.

## Conclusion

In conclusion, the LAM subdivisions were successfully and reproducibly identified on 3D TPUS data of vaginally nulliparous women. This paves the way for in vivo biomechanical analyses of the LAM which enables a better understanding of its (dys)function.

## Supplementary Information

**Additional File 1**. The 3D models of the four patients (a.-d.) not shown in the Figure 4 with the Pubic bone (PB, grey), the external sphincter (ES, brown), the puboperineal muscle (PPM, red), the puboanal muscle (PAM, orange), the pubovaginal muscle (PVM, yellow), the puborectal muscle (PRM, green) and the iliococcygeal muscle (ICM, blue). The view is simular to Figure 4a.

## Data Availability

Due to privacy regulations the data used in this study are not publicly available. In order to see and discuss the data the authors can be contacted. If needed, we can arrange approval to share the data with individual researchers.

## Abbreviations

3D/2D: 2/3 Dimensional
ATLA: Arcus tendineus levator ani
DSI: Dice similarity index
ES: External sphincter
ICM: Iliococcygeal muscle
LAM: Levator ani muscle
MRI: Magnetic resonance imaging
PAM: Puboanal muscle
PB: Pubic bone
PF: Pelvic floor
PPM: Puboperineal muscle
PRM: Puborectal muscle
PVM: Pubovaginal muscle
TPUS: Transperineal ultrasound
