# Supplementary material for: Appearance of the levator ani muscle subdivisions on 3D transperineal ultrasound
Source: Insights Imaging. 2021 Jul 2;12:91. doi: 10.1186/s13244-021-01037-y (PMC8253870; doi:10.1186/s13244-021-01037-y)

**Appendix 1**. 3D models of the four patients (A-D) not shown in the paper. Pubic bone (PB, grey), external sphincter (ES, brown), puboperineal muscle (PPM, red), puboanal muscle (PAM, orange), pubovaginal muscle (PVM, yellow), puborectal muscle (PRM, green) and iliococcygeal muscle (ICM, blue). As in figure 4A, the view from below is shown.


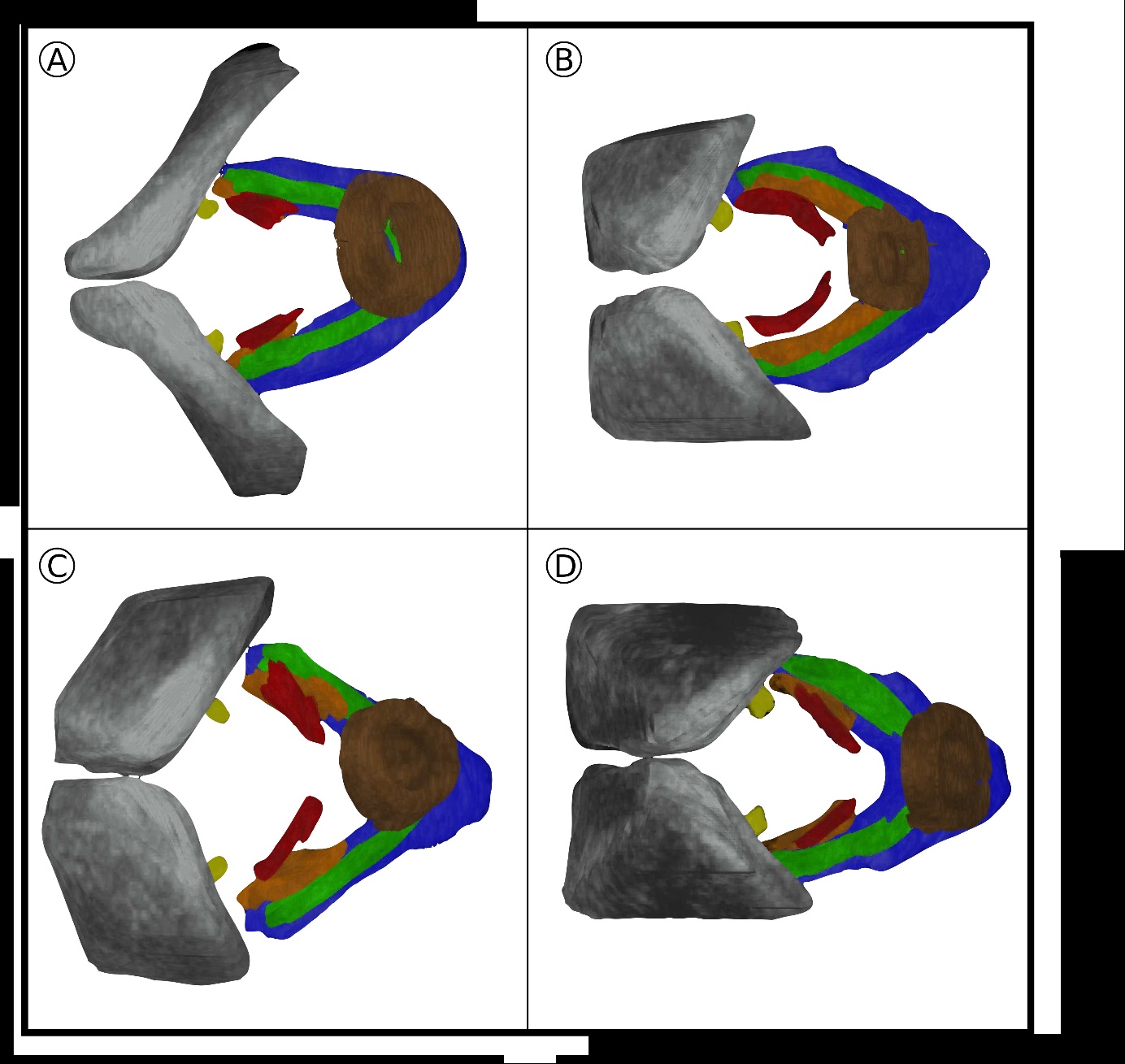

Supplement: Supplementary file 1 — Additional File 1. The 3D models of the four patients (a.-d.) not shown in the Figure 4 with the Pubic bone (PB, grey), the external sphincter (ES, brown), the puboperineal muscle (PPM, red), the puboanal muscle (PAM, orange), the pubovaginal muscle (PVM, yellow), the puborectal muscle (PRM, green) and the iliococcygeal muscle (ICM, blue). The view is simular to Figure 4a. [file 13244_2021_1037_MOESM1_ESM.docx]
